# Morquio's Syndrome: A Case Report of Two Siblings

**DOI:** 10.1155/2017/6176372

**Published:** 2017-01-16

**Authors:** Sathish Muthukumar Ramalingam, Daya Srinivasan, Sandhya ArunKumar, Joe Louis ChiriyanKandath, Sriram Kaliamoorthy

**Affiliations:** ^1^Department of Oral and Maxillofacial Pathology, Chettinad Dental College & Research Institute, Tamil Nadu, India; ^2^Department of Pedodontia and Preventive Dentistry, Chettinad Dental College & Research Institute, Tamil Nadu, India

## Abstract

Morquio syndrome or MPS IVA is a rare type of lysosomal storage disease associated with highly specific dental abnormalities. We present two siblings with enamel hypoplasia and skeletal abnormalities. A diagnosis of mucopolysaccharidosis type IVA was reached based on the clinical, radiographic, and dental findings of the patients. The dental findings are useful diagnostic aid for the early diagnosis of this debilitating disorder.

## 1. Introduction

Glycosaminoglycans (GAGs), formerly known as mucopolysaccharides, are long-chained carbohydrates that are vital for the formation of bone, cartilage, tendons, corneas, skin, and connective tissue. The deficiency of lysosomal enzymes required for the metabolism of the various mucopolysaccharides results in the accumulation of the respective substrate in the cells, leading to progressive cellular damage and clinic pathological changes. Mucopolysaccharidosis (MPS) is comprised of at least seven recognized phenotypes that are assigned by numbers and eponyms.

## 2. Case Report

**Figure 1:**
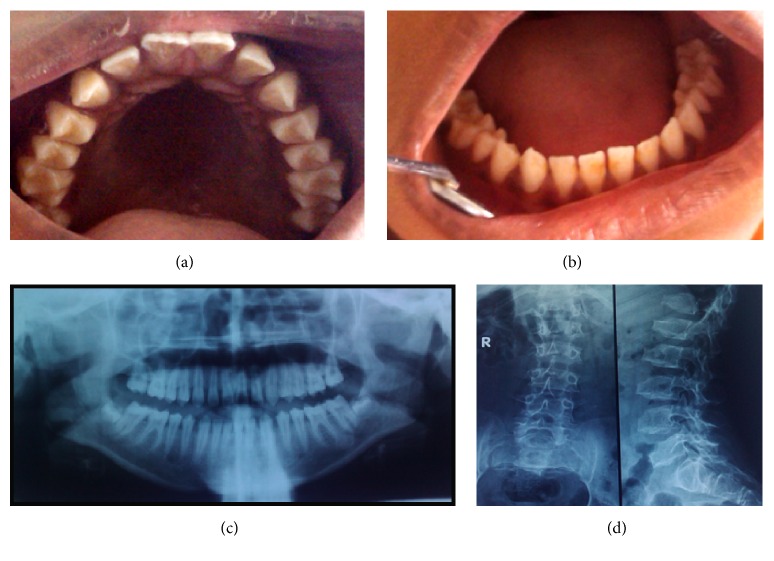
(a), (b) Spade-shaped incisors, pointed cusps, and spacing between teeth in case 1. (c) Orthopantomograph showing thin enamel with normal radio density. (d) Spinal radiographs revealing kyphoscoliosis and tongue-like projections from anterior surface of vertebral bodies in case 1.

**Figure 2:**
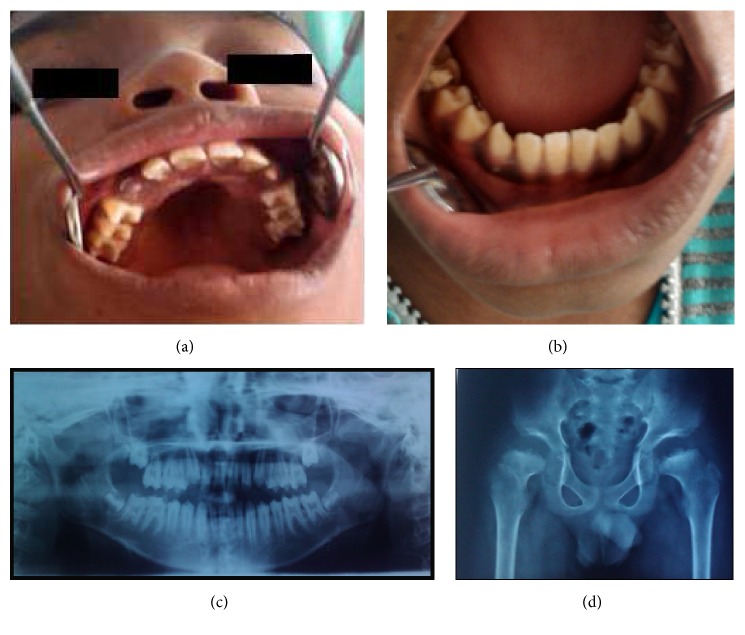
(a), (b) Spade-shaped incisors, pointed cusps, and spacing between teeth in both maxillary and mandibular teeth in case 2. (c) Orthopantomograph showing thin enamel with normal radio density. (d) Pelvic radiograph showing shallow acetabula and square-shaped femoral head in case 2.

Two siblings, a 13-year-old girl (case 1, Figures 1(a)–1(d)) and a 10-year-old boy (case 2, Figures 2(a)–2(d)) of normal intelligence had come to Chettinad Dental College and Research Institute, Chennai, with a complaint of decayed teeth. The physical appearances were similar, characterized by short stature, short neck, protuberant chest, scoliosis, and a waddling gait. Their facial feature presented frontal bossing and flat nasal bridge. The history revealed that the patients had pain in the hip region on walking that subsides at rest. The parents did not belong to different ethnicity. They had a nonconsanguineous marriage and a family history free from any skeletal disorder. On oral examination, exposed teeth with sharp cusps and rough enamel texture were found (Figures 1(a), 1(b), 2(a), and 2(b)). The orthopantomograph showed thin hypoplastic enamel of normal radiodensity (Figures 1(c) and 2(c)).

A multispecialty approach was adopted and medical examination of all major systems revealed no dysfunctional changes. However, the orthopedic and radiographic evaluation indicated gross skeletal deformity. The hand-wrist radiograph showed proximally narrow metacarpals. The anteroposterior view of the spine expressed curvature of the thoracolumbar vertebral segment. The lateral spinal view revealed anterior beaking of the vertebral body (Figure 1(d)). The pelvic view radiograph revealed an irregularly outlined square-shaped head of the femur and coxa valga defect (Figure 2(d)).

A diagnosis of mucopolysaccharidosis type IVA was reached based on the clinical, dental, and radiographic findings of the patients. The diagnosis would need to be confirmed by analysis of GALNS enzyme activity and molecular genetic testing of GALNS gene. Unfortunately, parents' consent could not be obtained for this analysis.

## 3. Discussion

Mucopolysaccharidosis type IV (MPS type IV) is a rare disorder which presents with a number of musculoskeletal defects. The clinical condition was first described independently in 1929 by Morquio and Brailsford. Matalon et al. in 1974 identified the deficiency of enzyme N-acetyl-galactosamine-6-sulfatase causing intracellular accumulation of keratan sulfate responsible for Morquio's syndrome type IVA [1]. MPS type IVB is due to deficiency of beta-galactosidase [2]. A non keratan sulfate excretion variety is labeled as MPS type IV C [3].

The parents did not belong to different ethnicity, yet they were not consanguineously related (first cousins, double first cousins, second cousins, double second cousin, or uncle niece relationship) [4]. Morquio patients usually do not present with any clinical signs or symptoms until the end of first year of life, but, as the age advances, they show signs of muscular atrophy, pectus carinatum, and knock knee deformity [5]. Obvious symptoms such as waddling gait and dwarfism due to dorsilumbar kyphoscoliosis can be observed at an earlier age [1]. Atlantoaxial subluxation and spinal cord compression especially in the upper cervical region are frequently noted. This is a sequela of odontoid dysplasia which is a major complication of MPS type IV [5, 6]. The later manifestations include corneal opacity, deafness, hypermobility of joints, cardiac abnormalities, quadriplegia and respiratory paralysis [7].

GAGs and proteoglycans provide the extracellular polyanionic macromolecules for the biomineralization process of several biologic systems. GAGs regulate the enamel maturation during the transitional and maturation stage of amelogenesis. Typical dental changes are characterized by surface pitting and thin hypoplastic enamel resulting in altered shape and discoloration [8]. The maxillary anterior teeth are widely spaced and the posteriors are occlusally tapered with pointed cusps [9, 10]. Enamel hypoplasia is a prominent feature in all forms of MPS type IVA. It is absent in MPS type IVB and MPSIVC. Thus, dental findings can be an important diagnostic aid for MPS type IVA [9–12]. GAGs serve as a matrix for anchoring amelogenin at the enamel dentinal junction so that a close bond is established between enamel and dentine [13]. Lack of sulfatase enzyme would remove the GAGS from dentinal tubule site which can cause lack of integration at enamel dentinal junction. Mineralization of enamel starts in the secretory stage of formation of enamel. Thickening of enamel crystallites to a preferred degree of orientation occurs in the maturation stage. Thus, texture of enamel is determined at both secretory and maturation stages. This could be the possible reason for rough enamel surface being noted [13, 14].

Inclination of the distal ends of the radius and ulna towards each other, hand bones being short, and squat help in radiological diagnosis. Convergence of proximal surfaces of metacarpals gives a “bullet-”shaped appearance [5–7]. Other findings which are observed in the present cases include coxa valga defect, odontoid hypoplasia, platyspondyly, and anterior beaking of the vertebral bodies. The pelvic deformity is characterized by short and square iliac wings associated with flat acetabula roof [5]. The anteroposterior spinal radiograph features thoracolumbar kyphoscoliosis as seen in the present case.

Diagnostic methods available are blood and urine analysis to quantify the keratan sulfate level, direct enzyme assay in leukocytes or fibroblasts, and the wide range of radiographic views to demonstrate the skeletal abnormalities. Although the elevated urinary keratan sulfate is diagnostic of MPS, mucopolysaccharide excretion reduces with age and cases of Morquio with absence of excessive keratan sulfate in the urine have also been reported [6, 7]. Odontoid hypoplasia or dysplasia and spinal cord compression are the most consistent features in Morquio's syndrome. A prenatal diagnosis for the disorder is possible by performing amniocentesis or chorionic villi sampling when there is a family history of Morquio's syndrome. The diagnosis has to be confirmed with GALNS enzyme activity and molecular genetic testing of GALNS gene [15]. In the present case, the children are born out of a nonconsanguineous marriage and no family members are affected. The final diagnosis is based on clinical, radiographic, and dental findings.

In conclusion, MPS IVA is a rare type of mucopolysaccharidosis associated with highly specific dental abnormalities. As there is no cure for Morquio's syndrome, periodic monitoring and intervention are mandatory. Bone marrow transplant and enzyme replacement therapy (ERT) have been used with some success. This case illustrates the importance of systemic evaluation and inclusion of MPS type IVA in the differential diagnosis of enamel hypoplasia. As both the primary and permanent dentitions are affected, an early diagnosis is possible even in the clinically atypical cases. Early systemic evaluation and follow-up will help in improving the quality of life of the patients.
